# Supplementary material for: Content-rich biological network constructed by mining PubMed abstracts
Source: BMC Bioinformatics. 2004 Oct 8;5:147. doi: 10.1186/1471-2105-5-147 (PMC528731; doi:10.1186/1471-2105-5-147)
Supplement: Additional File 2 — The original results of the above study (non-essential files are deleted to keep the file size under the limit set by BMC bioinformatics). [file 1471-2105-5-147-S2.bz2 › chilibotAdditionalFile2/dip20/36ID7539137E131/html/FKBP1A_FRAP1.html]

 


 **FKBP1A** and **FRAP1** 
  
Found 77 abstracts in PubMed, retrieved 20.  
 

 What does Google say? 
 PDF only 
| .edu only 

---

**Interactive relationship** (e.g. stimulation, inhibition, etc)

**Inhibitory relationship**- Rapamycin FKBP12  [ **FKBP1A** ]  inhibited mTOR  [ **FRAP1** ]  activity.  Ref: 12370290 Mol Cell Biol, 2002
- First, mutations of mTOR  [ **FRAP1** ]  or FKBP12  [ **FKBP1A** ]  prevent rapamycin from binding to mTOR  [ **FRAP1** ] , conferring rapamycin resistance.  Ref: 12030785 Drug Resist Updat, 2001
- In essence, these agents gain function by binding to the immunophilin FK506 binding protein 12  [ **FKBP1A** ]  and the resultant complex inhibits the activity of mTOR  [ **FRAP1** ] .  Ref: 12864941 Clin Breast Cancer, 2003
**Neutral relationship**- Capture of the long lived interaction complex allows the isolation of phage bearing cognate interaction pairs, as we demonstrate for a range of interactions, including Ab antigen pairs and the rapamycin dependent interaction of FKBP 12  [ **FKBP1A** ]  and FRAP  [ **FRAP1** ] .  Ref: 12084913 Proc Natl Acad Sci U S A, 2002
- The FKBP 12  [ **FKBP1A** ]  rapamycin associated protein FRAP  [ **FRAP1** ] , also known as mTOR  [ **FRAP1** ]  and RAFT 1  [ **FRAP1** ]  is a member of the phosphoinositide kinase related kinase family.  Ref: 11707573 Proc Natl Acad Sci U S A, 2001
- FKBP12  [ **FKBP1A** ]  rapamycin associated protein FRAP  [ **FRAP1** ] , also known as mTOR  [ **FRAP1** ]  or RAFT  [ **FRAP1** ]  is the founding member of the phosphatidylinositol kinase related kinase family and functions as a sensor of physiological signals that regulate cell growth.  Ref: 11930000 Proc Natl Acad Sci U S A, 2002
- To effect membrane recruitment, a myristoylated rapamycin binding domain from FRAP  [ **FRAP1** ]  mTOR  [ **FRAP1** ] , called M FRB, binds to lipid permeable rapamycin and non bioactive synthetic rapalogs, leading to reversible heterodimerization of M FRB with FKBP  [ **FKBP1A** ]  DeltaPH.Akt.  Ref: 11896462 Gene Ther, 2002

**Non-interactive relationship** (e.g. studied together, co-existance, homology, etc.)

- Syncytium induced p53S15 phosphorylation is mediated by the upregulation activation of mammalian target of rapamycin  [ **FRAP1** ]  mTOR  [ **FRAP1** ] , also called FKBP12  [ **FKBP1A** ]  rapamycin associated protein FRAP  [ **FRAP1** ] , which coimmunoprecipitates with p53.  Ref: 11602639 J Exp Med, 2001
- The FKBP12  [ **FKBP1A** ]  rapamycin associated protein FRAP  [ **FRAP1** ]  is a CLIP 170 kinase.  Ref: 12231510 EMBO Rep, 2002
- In complex with its cellular receptor, the FK506 binding protein FKBP12  [ **FKBP1A** ] , rapamycin binds and inhibits the function of the mammalian target of rapamycin  [ **FRAP1** ]  mTOR  [ **FRAP1** ] .  Ref: 12234610 Biochem Pharmacol, 2002
- Rapamycin binds intracellularly to the immunophilin FK506 binding protein 12  [ **FKBP1A** ]  FKBP12  [ **FKBP1A** ] , and the resultant complex inhibits the protein kinase activity of a protein kinase termed mammalian target of rapamycin  [ **FRAP1** ]  mTOR  [ **FRAP1** ] .  Ref: 11426655 Oncogene, 2000
- In this study, we show that FKBP12  [ **FKBP1A** ]  rapamycin associated protein FRAP  [ **FRAP1** ] , also called mTOR  [ **FRAP1** ]  RAFT  [ **FRAP1** ]  interacts with CLIP 170.  Ref: 12231510 EMBO Rep, 2002
- Upon BMP4 treatment, the serine threonine kinase FKBP12  [ **FKBP1A** ]  rapamycin associated protein FRAP  [ **FRAP1** ] , mammalian target of rapamycin  [ **FRAP1** ]  mTOR  [ **FRAP1** ] , associates with Stat3 and facilitates STAT activation.  Ref: 12796477 J Cell Biol, 2003
- In the full length enzyme these sites are also acutely sensitive to the action of FKBP 12  [ **FKBP1A** ]  rapamycin associated protein FRAP  [ **FRAP1** ] .  Ref: 11684675 J Biol Chem, 2002
- The FK506 FKBP12  [ **FKBP1A** ]  binding domain of the kinase FRAP  [ **FRAP1** ]  FRB forms a classic up down four helical bundle.  Ref: 12175232 J Am Chem Soc, 2002
- Rapamycin inhibits the FK506 binding protein 12  [ **FKBP1A** ]  mammalian target of rapamycin  [ **FRAP1** ]  complex and causes cell cycle arrest in G1.  Ref: 12599018 Urol Res, 2003
- Human immunodeficiency virus 1 envelope glycoprotein complex induced apoptosis involves mammalian target of rapamycin  [ **FRAP1** ]  FKBP12  [ **FKBP1A** ]  rapamycin associated protein mediated p53 phosphorylation.  Ref: 11602639 J Exp Med, 2001
- Such activation of p70 S6K is blocked by pharmacological inhibitors of the PI 3 kinase or the FKBP 12  [ **FKBP1A** ]  rapamycin associated protein mammalian target of rapamycin  [ **FRAP1** ]  FRAP  [ **FRAP1** ]  mTOR  [ **FRAP1** ] .  Ref: 12759354 J Biol Chem< MedlineTA>J Biol Chem, 2001
- The signal from DNA damage to activation of these response pathways appears to be mediated by FKBP12  [ **FKBP1A** ]  rapamycin associated protein FRAP  [ **FRAP1** ] , since these phosphorylation events are blocked by rapamycin.  Ref: 12231188 Methods, 2002
- Rapamycins first bind a cyclophilin FKBP12  [ **FKBP1A** ] , and this complex binds and inhibits the function of mTOR  [ **FRAP1** ]  mammalian target of rapamycin  [ **FRAP1** ]  a serine threonine Ser Thr kinase with homology to phosphatidylinositol 3 kinase.  Ref: 12878853 Cancer Biol TherCancer Biol Ther, 2002
- eIF4G Delta and Delta eIF4G were fused C and N terminally to the FK506 binding protein FKBP  [ **FKBP1A** ]  and the FKBP  [ **FKBP1A** ]  rapamycin binding domain FRB of the human FKBP  [ **FKBP1A** ]  rapamycin associated protein FRAP  [ **FRAP1** ] , respectively.  Ref: 12768627 Biotechnol Bioeng, 2003
